# Recruitment and retention of participants in a pragmatic randomized intervention trial at three community health clinics: Results and lessons learned

**DOI:** 10.1186/1471-2458-13-192

**Published:** 2013-03-06

**Authors:** Erica T Warner, Russell E Glasgow, Karen M Emmons, Gary G Bennett, Sandy Askew, Bernard Rosner, Graham A Colditz

**Affiliations:** 1 Department of Epidemiology, Harvard School of Public Health, 677 Huntington Ave, Boston, MA, USA; 2 Center for Community-Based Research, Division of Population Sciences, Dana Farber Cancer Institute, 375 Longwood Ave, Boston, MA, USA; 3 Channing Division of Network Medicine, Brigham and Women’s Hospital, 181 Longwood Ave, Boston, MA, USA; 4 Division of Cancer Control and Population Sciences, National Cancer Institute, 6130 Executive Boulevard, Executive Plaza North, Rockville, MD, USA; 5 Department of Society, Human Development, and Health, Harvard School of Public Health, 677 Huntington Ave, Boston, MA, USA; 6 Department of Psychology and Neuroscience, Duke University, Box 90086, Durham, NC, USA; 7 Duke Global Health Institute, Duke University, Box 90086, Durham, NC, USA; 8 Department of Biostatistics, Harvard School of Public Health, 677 Huntington Ave., Boston, MA, USA; 9 Department of Medicine, Harvard Medical School, 25 Shattuck St., Boston, MA, USA; 10 Department of Surgery, Alvin J. Siteman Cancer Center, Washington University School of Medicine, Saint Louis, MO, USA

**Keywords:** Health disparities, Weight-loss, Obesity, Hypertension, Pragmatic trial, Recruitment, Retention

## Abstract

**Background:**

Obesity and hypertension and their associated health complications disproportionately affect communities of color and people of lower socioeconomic status. Recruitment and retention of these populations in research trials, and retention in weight loss trials has been an ongoing challenge.

**Methods:**

Be Fit, Be Well was a pragmatic randomized weight loss and hypertension management trial of patients attending one of three community health centers in Boston, Massachusetts. Participants were asked to complete follow-up assessments every 6-months for two years. We describe challenges encountered and strategies implemented to recruit and retain trial participants over the 24-month intervention. We also identify baseline participant characteristics associated with retention status. Retention strategies included financial incentives, contact between assessment visits, building relationships with health center primary care providers (PCPs) and staff, and putting participant convenience first.

**Results:**

Active refusal rates were low with 130 of 2,631 patients refusing participation (4.9%). Of 474 eligible persons completing telephone screening, 365 (77.0%) completed their baseline visit and were randomized into the study. The study population was predominantly non-Hispanic Black (71.2%), female (68.5%) and reported annual household income of less than $35,000 (70.1%). Recruitment strategies included use of passive approval of potential participants by PCPs, use of part-time staff, and outsourcing calls to a call center. A total of 314 (86.0%) people completed the 24-month visit. Retention levels varied across study visits and intervention condition. Most participants completed three or more visits (69.6%), with 205 (56.2%) completing all four. At 24-months, lower retention was observed for males and the intervention condition. Retention strategies included building strong relationships with clinic staff, flexibility in overcoming participant barriers through use of taxi vouchers, night and weekend appointments, and keeping participants engaged via newsletters and social gatherings.

**Conclusion:**

We were able to retain 86.0% of participants at 24-months. Recruitment and retention of high percentages of racial/ethnic minorities and lower income samples is possible with planning, coordination with a trusted community setting and staff (e.g. community health centers and RAs), adaptability and building strong relationships.

**Trial registration:**

Clinicaltrials.gov Identifier: NCT00661817

## Background

Recruitment and retention of racial/ethnic minorities, particularly non-Hispanic Blacks (Blacks) and Hispanics, into clinical trials continues to be a challenge. A distrust of the medical community due to historical injustices such as the Tuskegee Syphilis Study is an oft cited reason for low participation [1-4]. Yet other factors may lead to exclusion from efficacy trials, such as a lack of trials occurring in racial/ethnic minority serving institutions, limited awareness of ongoing trials, work/family and insurance barriers, trial referral practices of PCPs, and the prevalence of co-morbid conditions [5-7]. Regardless of the challenges, inclusion of these groups in clinical research is important to ensure that results are relevant, and generalizable [8]. Many of the health conditions under study in trials disproportionately affect communities of color, and thus their participation is critical [9].

Weight loss interventions have generally been less successful among Blacks. For example, the Trial of Non-pharmacologic Interventions in the Elderly found that Blacks lost on average 2.9 kilograms less than Whites over the course of the intervention [10]. Similar disparities were seen in the lifestyle treatment arm of the Diabetes Prevention Program. At 24-months weight loss was lower among black men (−5.9%) and women (−3.2%) than White men (−7.2%) and women (−5.9%) or Hispanic men (−7.8%) and women (−7.1%) [11]. Yet, rates of obesity and obesity-related conditions are very high among Black and Hispanic populations. According to data from the National Health and Nutrition Examination Study, 44.1% of Blacks were obese compared with 38.7% of Hispanics and 32.4% of non-Hispanic Whites (Whites) [12]. Rates of obesity-related conditions such as diabetes, high blood pressure, and asthma are higher among Blacks and Hispanics than among Whites [13]. There is clearly a need for interventions that are effective at weight loss and weight maintenance among Black and Hispanic populations. In order to design and implement interventions that will work for this population, we need well-designed trials with high representation of these at-risk groups. Thus increasing participation and effectiveness among Black and Hispanic populations in weight loss studies is of great importance.

Long-term weight loss trials have traditionally had low to moderate retention levels. For example, the Louisiana Obese Subjects Study reported retention rates at 24-months of 51% in their intervention group and 46% in usual care [14]. While several studies such as the Trials of Hypertension Prevention have had high levels of retention [15], a review of 16 weight loss trials showed that nine had relatively high (31–64%) levels of loss to follow-up [16]. Study interpretability declines with increasing levels of loss to follow-up in intervention trials [16-18]. Therefore, it behooves us to understand strategies that yield high retention levels.

This report describes the recruitment and retention of participants in a pragmatic randomized weight loss and hypertension management trial, ‘Be Fit, Be Well’ [19,20]. The purposes of this paper are to: (1) describe recruitment and retention activities; (2) discuss challenges encountered and strategies used to overcome them; (3) analyze study retention and patterns of retention and its association with baseline participant characteristics, and (4) describe lessons learned and directions for future research.

## Methods

Be Fit, Be Well (BFBW) was a randomized, controlled, intervention trial funded by the National Heart, Lung and Blood Institute under a U01 grant mechanism. Participants were recruited from three community health clinics in the Boston neighborhoods of Roxbury and Dorchester. Primary eligibility criteria were age 21 or older, a body mass index (BMI) between 30 and 50 kg/m^2^, and diagnosed hypertension with prescribed anti-hypertensive medication.

Exclusion criteria have been described elsewhere but, included: a history of myocardial infarction, stroke, or an atherosclerotic cardiovascular disease procedure ≤ 6 months before study entry, prior or planned bariatric surgery, recent or planned pregnancy, use of Food and Drug Administration (FDA) approved weight loss medications or medications known to increase weight [21,22].

At baseline all participants had their weight, height and blood pressure measured, received the National Heart Lung and Blood Institute’s *Aim for a Healthy Weight* brochure, and completed an audio computer assisted self-interview survey which collected data on demographics, health behaviors, mental health and neighborhood characteristics. Participants randomly assigned to the usual care group received no further educational materials or instruction throughout the study period, but returned for follow-up assessments every 6 months. The study intervention is described elsewhere in greater detail [22]. Briefly, intervention participants received materials for behavior self-monitoring including a pedometer, 18 attempted calls from a community health educator (monthly in year 1, bi-monthly in year 2), were invited to attend optional monthly group sessions and received information about community resources. In concert with their assigned community health worker (CHW), intervention participants set behavioral goals, tracked them via their selected modality (website or interactive voice response system), received tailored feedback on their progress and had the opportunity to modify their goals every 13 weeks. All study procedures, materials and modifications were approved by the Institutional Review Board at Harvard School of Public Health (protocol #14455).

All participants were asked for their preferred language and study materials were available in English and Spanish. A BFBW study logo was on all printed materials, including letterhead, envelopes and other mailings. The study had a dedicated email address and two dedicated landlines. All participants were asked to complete follow-up data collection visits every six months for two years. At follow-up visits participants had their weight and blood pressure measured and completed a computer-assisted survey. Baseline and follow-up visits were conducted by trained, bilingual RAs (RAs) and were generally less than one hour in length. As there were two RAs and three health clinics, each RA had primary responsibility for one health clinic and shared responsibility for participants at the third. A part-time project director oversaw the work of the RAs and coordinated with health clinic staff and medical directors.

### Recruitment protocol

We used primarily active recruitment methods [23]. Potentially eligible patients were identified largely through a two-step process of preliminary medical record searches using the primary eligibility criteria of obesity and hypertension, followed by more thorough medical record review. At the two clinics with electronic medical records (EMR), clinic staff provided lists of patients with diagnosed hypertension (using ICD-9 codes) and obesity (using measured height and weight). The clinic without an EMR was able to provide a list of people with ICD-9 codes for hypertension using their claims billing system. Trained RAs performed medical record reviews on each identified patient to assess study eligibility.

Lists of potentially eligible patients were sent to their PCP for confirmation and permission to contact. RAs contacted approved patients by mail. Letters explained the study, told patients to expect a call in the next five days, and were printed on clinic letterhead with the signature of the patient’s PCP. RAs called and administered a brief eligibility questionnaire and answered any questions. Calls were tracked using call logs. Up to 10 phone calls were allocated per potential participant. Eligible and interested patients that could be reached were scheduled for a baseline study visit at their clinic.

Appointments were monitored using Google calendar using a unique ID for each potential participant. The project director generated weekly recruitment status reports which aggregated data on the number of medical records reviewed, potentially eligible patients identified, names submitted to PCPs for approval, intro letters mailed, eligibility calls made/completed, number of appointments scheduled and number of participants randomized. Study staff discussed these weekly reports and developed improvement strategies throughout the recruitment period as challenges arose. We aimed to enroll 360 participants over the course of one year. We anticipated enrollment to be approximately 66% black and 30% Hispanic.

### Retention protocol

Participants scheduled their next follow-up visit appointment at the end of each completed follow-up visit (i.e. 6-month visit scheduled at end of baseline visit). If a visit was missed, the RAs called by phone and/or sent letters and emails to schedule their next appointment. Each participant appointment was identified using their participant identification number and appointment outcomes were updated daily via Google calendar. Visit outcomes were classified as: completed, rescheduled, or no show. Distinction was made between appointments rescheduled for study or personnel reasons versus appointments that were rescheduled by the participant’s request. Completed follow-up appointments were classified as within or out of window. The visit window was calculated as six weeks before and after the visit date as calculated based on their baseline visit.

At the baseline visit we asked for home, work and cell phone numbers of each participant and the name and number of two people who we could contact in the event that we could not reach them at any of the other numbers provided [24]. Reminder letters were sent two weeks before each scheduled visit and reminder calls were made one week prior and the day of each visit. In the event of a missed appointment, we attempted to reschedule the visit via phone, email (where available) and/or mailed letter.

We offered flexible visit scheduling; both in time (evening and Saturday visits) and location (could come to any of the three health clinics). We mailed birthday cards to all participants in their preferred language. Participants received incentives to compensate them for their time at baseline and follow-up visits [25]. Upon completion of baseline, 6-, 12, and 18-month visits participants received a $50 grocery card and at 24-months they received a $75 grocery card. Intervention participants also received a scale at their 12-month visit and a blood pressure monitor at 18-months to support their behavioral self-monitoring goals.

For each participant and each follow-up visit we calculated expected visit date, date visit window opened, date visit window closed, logged whether the visit was completed, visit completion date, and made notes about the participant’s status with the study and our attempts at contact. This information was organized into spreadsheets that were updated on a weekly basis, cross-referenced with the Google calendar and reviewed at weekly team meetings to assure accuracy. Participants that were significantly out of window for a given follow-up visit were classified as missed for that visit and contacted for completion of their next visit. Retention percentages were monitored weekly and new strategies developed and implemented as needed. IRB amendments were submitted Participants did not directly participate in strategy development, but they were informally queried about reasons for participation and barriers. The information they provided did inform our strategies.

### Statistical analyses

Logistic regression was used to generate multivariable adjusted p-values comparing the probability of retention at each follow-up visit (6-, 12-, 18- or 24-months) across categories of baseline characteristics including randomization group, age, gender, race, income, primary language, depression score, education, work status and self-rated health. All p-values are two-sided with an alpha level 0.05. All analyses were conducted using SAS 9.2.

## Results

### Participant recruitment

**Figure 1:**
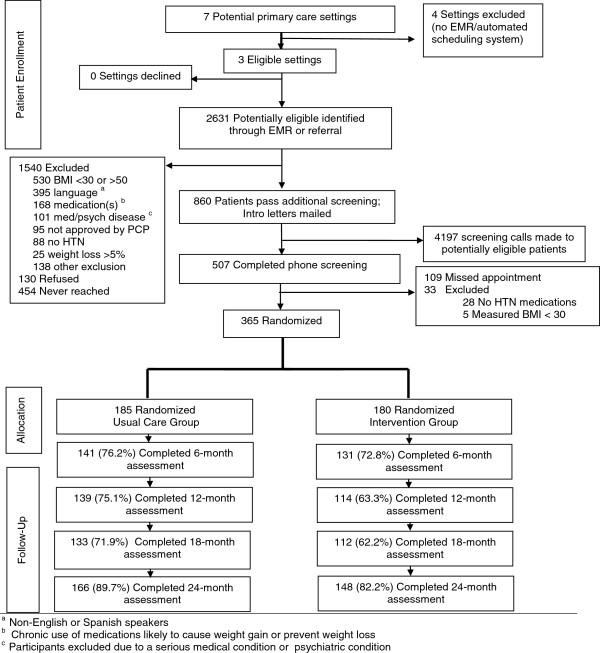
CONSORT flow diagram of health center selection, participant screening, eligibility and retention: Be Fit, Be Well Study.

We reviewed over 2500 medical records, mailed 860 introductory letters, and made over 4100 eligibility screening call attempts (Figure 1). Less than 5% of potentially eligible participants actively refused study enrollment (130 of 2631). Few people that completed our eligibility screening call (33 of 507 (6.5%)) were deemed ineligible; 365 (77%) of those eligible and with scheduled baseline visit appointments completed their baseline visit and were randomized. Of the 109 people that did not complete their baseline visit, 48 (44.0%) were male, 28 (25.7%) had a Spanish surname and mean age was 51.5 years (data not shown). We enrolled our last participant on April 30, 2009 for a total recruitment period of 15 months.

**Figure 2:**
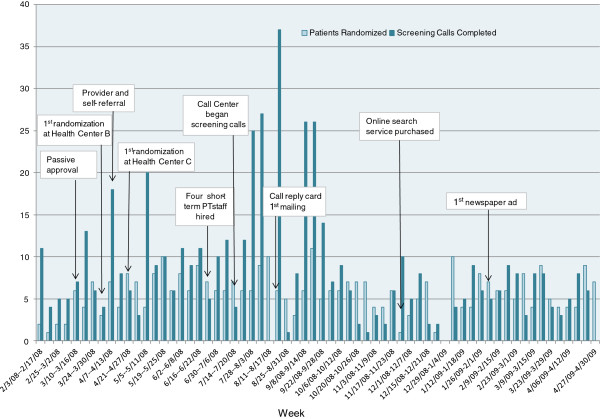
Weekly number of screening calls completed and randomized participants by implementation date of recruitment strategies.

Figure 2 displays the number screening calls completed and participants recruited per week and includes when strategies were implemented. Most strategies were implemented during the first six months of the recruitment period, making it difficult to disentangle their effects. Passive PCP approval’s implementation one month after the start of recruitment increased the pool of potentially eligible participants we could contact. Of our other strategies, hiring additional short-term part-time staff and use of the call center greatly increased our capacity complete screening calls and randomize participants. One month after implementing the call center, our weekly number of screening calls completed was nearly 7 times greater than the week they started (Figure 1). However, the reply card mailing, PCP and patient self-referral, and newspaper ads were not incrementally effective (Table 1). PCPs rarely used the clinic drop boxes to submit patients for the study. Only one person that responded to our newspaper ads went on to be randomized.

**Table 1:** Be Fit, Be Well recruitment strategies and results

***Protocol***	***Challenge*( *s *)**	***Revised strategy***	***Result***
Identify potentially eligible participants through medical record review	• Difficult to maintain large pool of potential participants for contact	1. Provider referral: providers could submit participants via faxed or emailed referral form; drop boxes for referrals placed in	1. Received less than 20 provider referrals. Most were people already identified through medical record review;
		2. Patient self-referral: flyers were placed in waiting and patient rooms with brief study description and contact information;	2. Received no inquiries from patient self-referral
		3. Refer-a-friend: enrolled participants were asked to tell their friends about the study;	3. Received less than 10 suggestions from enrolled participants. Most were ineligible.
		4. Newspaper ads: Two ads were run in a publication that was distributed to people riding public transportation; one in paper serving the African-American community; Another in a Spanish language paper serving the Hispanic/Latino community	4. Received less than 20 inquiries from newspaper ads. Most were not patients at one of the three health centers and were ineligible. One person was ultimately enrolled.
			>95% of enrolled participants were identified via medical record review
Participant names submitted to their primary medical provider for approval to contact for study enrollment	• Long delays in receipt of provider approval	1. Implemented passive provider approval system. We divided patients into ‘Needs Confirmation’ and ‘Confirmed’ groups. Based on medical record review, patients in the ‘Confirmed’ group met all eligibility criteria and were free of diabetes, CVD and peripheral vascular disease. Providers had the option to exclude patients from this list, but we initiated patient contact after 10 business days if providers had not responded. The ‘Needs Confirmation’ group included people with at least one of the aforementioned health conditions. This group still required explicit approval from providers before contact.	1. Prior to passive approval, we had submitted 431 names to providers and had received a response on 212 (49.1%) of them. With passive approval in place, there were just 29 still awaiting provider approval at the end of recruitment. Providers also expressed appreciation for the passive process that, given their demanding schedules, reduced their study related workload considerably.
			Passive approval decreased time from patient identification to initial contact significantly.
Approved participants sent introductory letter and called by research assistants to confirm eligibility using contact information in medical records	• Incorrect contact information;	1. Collaborated with health center administrative staff to obtain regular updates of patient contact information;	1. Health center staff generally did not have more up to date contact information than what they had originally provided us;
	Large number of calls (> 4000) required to garner each scheduled baseline visit	2. Purchased subscription to online people search website to find new addresses and phone numbers for potential participants;	2. Website provide correct contact information for some potential participants;
		3. Mailed potentially eligible participants a self-addressed, postage-paid card with our contact phone number which requested the three best times to call, best phone number to use and phone number type (home, work, cell), any alternate phone numbers. Card completion qualified them for a $75 Target gift card raffle.	3. Few cards were returned among participants with incorrect phone numbers. Many were returned to sender as incorrect phone numbers were highly correlated with incorrect mailing addresses.
		4. Hired an off-site call center to make intake calls. Trained survey assistants at the University of Massachusetts Amherst Survey Research Center made all English calls. Research assistants continued to make calls in Spanish.	4. Call center improved the weekly call volume and allowed the research assistants to focus on other recruitment tasks.
			454 (17%) potentially eligible participants had never been reached at the end of recruitment.
Two research assistants responsible for completion of all baseline and follow-up assessments	• Baseline and follow-up visits occurring simultaneously	1. Hired and trained short-term part-time staff to help with completion of baseline and follow-up visits	1. Seven short-term staff were largely students hired through institutional internship programs. Generally there were only two working during any given time period, but there was one 3-month period with four. Each short-term staff member underwent training and certification in measuring height, weight and blood pressure, shadowed the full-time RAs for 2–3 visits, and led their first three visits with a full-time RA present to ensure data quality.
			They greatly facilitated completion of visits, particularly during a point when baseline, 6-month and 12-month visits were all ongoing.

### Participant retention

Table 2 summarizes retention strategies employed and their results. The most useful activities in terms of response from participants, and our administrative assessment, were regular communication with clinic staff and PCPs, newsletters and social gatherings for participants and extra incentives to participants with previous missed visits. CHWs were also helpful in scheduling appointments with active intervention participants.

**Table 2:** Be Fit, Be Well retention strategies and results

***Protocol***	***Challenge*( *s *)**	***Revised strategy***	***Result***
RAs call participants from their assigned health center two weeks before an upcoming follow-up appointment, one-week before and the day of to confirm. Calls made during day, evenings and weekends to home, cell and work numbers	• Unable to reach participant	1. Prepaid phones for research assistants to facilitate evening and weekend calls;	1. RA night and weekend calls increased with use of prepaid phones;
		2. RAs called participants not from their primary health center;	2. Calls to participants from different research assistants were not effective
		3. $5 Gift card mailing to request updated contact information;	3. $5 gift card mailings yielded few updates to contact information. Most of the people that responded were people for whom we already had correct information;
		4. Surveyed participants on economic hardships at 24-month visit to understand the role this may have played;	4. Of the 144 participants surveyed, 39 (27.3%) said their phone had been disconnected in the past 24-months.
RAs attempt to contact participants with missed appointments by phone, email and mail.	• Repeated missed appointments	1. RAs met patient at clinic when scheduled to see their provider and measure weight only	1. Difficult to coordinate schedules to be at clinic for patient doctor appointments; Some patients not keeping appointments with BFBW were also not seeing their provider.
	Missed follow-up visits for participants inactive in the intervention arm	2. Primary care provider reengagement message;	2. Provider reengagement messages were not consistently delivered to participants. Providers found it hard to keep track of who was in need of messaging;
		3. Offered gift cards from missed visits as incentive to complete last visit;	3. Of the 158 people eligible to receive gift cards from missed visits, 108 completed their 24-month visit; 52 of those completions occurred after the mailing;
		4. Taxi vouchers;	4. Taxi vouchers were used by less than 20 participants overall. Those using vouchers tended to be elderly and were generally reliant on family members for transportation;
		5. Home visits at 24-months only	5. Completed a total of five home visits. In response to our offer of coming to their home, several people said things like, ‘You don’t have to go to all that trouble. I can come to the clinic.’ Several of these people did complete their visit at the clinic.
		6. Use any clinic measured weight within three months of the scheduled assessment date	6. We were able to get clinic weights for several participants. However, many patients that missed appointments with BFBW also were not seeing their PCP.
		7. Community health workers (CHW) help with scheduling.	7. Among those intervention participants in contact with their CHW, having the CHW give reminders about upcoming follow-up visits and help reschedule missed visits was successful.
Maintain participant contact with holiday and birthday cards	Disengagement among participants	1. Each quarter we sent out a newsletter with our contact information, updates on the study and an appeal to complete study visits to all participants. It included health articles like how to avoid overindulging during the holidays or ways to get out and enjoy Boston in the spring.	1. Quarterly newsletters were well received and occasionally prompted a phone call from a participant trying to find out if they were supposed to come in for a follow-up visit soon;
		2. Social gatherings were held quarterly. To prevent unblinding, only intervention participants and control participants that had completed the program were invited. We encouraged to bringing family and friends.	2. We held four social gatherings and each had between 25–50 attendees. Those that came reported that they enjoyed themselves.
Follow-up visits occur at health centers in assigned BFBW space or in available patient exam rooms	Difficult to maintain consistent space for follow-up visits at health centers	1. Space administrators were given a copy of our visit schedule a week in advance for space planning. Front desk staff was provided with a BFBW info card and the RA contact information. Each day they received a list of expected participants. We placed removable placards on the door of our visit space that announced that it was in use by BFBW and included a schedule for the day.	1. Communication with staff on the part of the project director and the research assistants was essential. These relationships greatly facilitated our ability to complete follow-up visits at the clinics.

Completion percentages varied by visit, with the highest retention observed at 24- (86.0%) and 6-months (74.5%) and the lowest at 12- (69.3%) and 18-months (67.1%) (Table 3). At 6-months lower retention was observed for those with income less than $10,000 (64.2%) compared to those with income of $35,000 or more (80.7%). Retention was consistently, but non-significantly, higher at Health Center A as compared to the other two sites. At 12 months, retention at Health Center B (62.1%) was significantly lower than that observed Health Center A (75.3%; p=0.01). There was also higher retention of the usual care group compared to those in the intervention which was statistically significant at 12- (75.1% vs. 63.3%; p=.00039) 18-(71.9% vs. 62.2%; p=0.01) and 24-months (89.7% vs. 82.2%; p=0.03), but not at 6 months (p=0.19). Persons under age 50 at baseline had lower retention than older persons at 6-, 12- and 18-month visits. At 18 months, women (71.2%) had higher retention than men (58.3%; p=0.04). A similar pattern for gender was seen at 24-months. At the 18-month visit, we observed lower retention of Hispanic participants (47.9%) as compared to Blacks (70.4%; p=0.03). The other characteristics examined including educational attainment, depression score, self-rated health, and language were not associated with retention at any study visit.

**Table 3:** Retention percentages by baseline participant characteristics in Be Fit, Be Well trial

	***Baseline N *(%)**	***6*- *month N *(%)**	***12*- *month N *(%)**	***18-month N*(%)**	***24-month N*(%)**
**Overall**	365 (100.0)	272 (74.5)	253 (69.3)	245(67.1)	314 (86.0)
**Group**					
Usual care	185 (50.7)	141 (76.2)	139 (75.1)**	133 (71.9)*	166 (89.7)*
Intervention	180 (49.3)	131 (72.8)	114 (63.3)	112 (62.2)	148 (82.2)
**Age (years)**					
<50	97 (26.6)	66 (68.0)*	58 (59.8)*	51 (52.6)**	79 (81.4)
50-59	91 (24.9)	70 (76.9)	68 (74.7)	65 (71.4)	81 (89.0)
60-69	93 (25.4)	75 (80.7)	72 (77.4)	74 (79.6)	81 (87.1)
≥ 70	84 (23.0)	61 (72.6)	55 (65.5)	55 (65.5)	73 (86.9)
**Gender**					
Female	250 (68.5)	186 (74.4)	177 (70.8)	178 (71.2)*	222 (88.8)*
Male	115 (31.5)	86 (74.8)	76 (66.1)	67 (58.3)	92 (80.0)
**Race/Ethnicity**					
Black	260 (71.2)	198 (76.2)	187 (71.9)	183 (70.4)	225 (86.5)
Hispanic	48 (13.2)	35 (72.9)	32 (66.7)	23 (47.9)*	40 (83.3)
White or other	57 (15.6)	39 (68.4)	34 (59.7)	39 (68.4)	49 (86.0)
**Language**					
English	318 (87.1)	238 (74.8)	220 (69.2)	217 (68.2)	274 (86.2)
Spanish	47 (12.9)	34 (72.3)	33 (70.2)	28 (59.6)	40 (85.1)
**Health center**					
A	158 (43.3)	122 (77.2)	119 (75.3)	116 (73.4)	138 (87.3)
B	103 (28.2)	77 (74.6)	64 (62.1)*	65 (63.1)	87 (84.5)
C	104 (28.5)	73 (70.2)	70 (67.3)	64 (61.5)	89 (85.6)
**Education**					
< High school	120 (32.9)	83 (69.2)	85 (70.8)	76 (63.3)	102 (85.0)
High school graduate	109 (29.9)	79 (72.5)	71 (65.1)	71(65.1)	97 (89.0)
Some college or college graduate	136 (37.3)	110 (80.9)	97 (71.3)	98 (72.1)	115 (84.6)
**Work status**					
Employed or student	196 (53.7)	149 (76.0)	133 (67.9)	131 (66.8)	169 (86.2)
Unemployed or disabled	110 (30.1)	83 (75.5)	83 (75.5)*	78 (70.9)	95 (86.4)
Homemaker or retired	59 (16.2)	40 (67.8)	37 (62.7)	36 (61.0)	50 (84.8)
**Annual income (in $)**					
<10,000	95 (26.0)	61 (64.2)*	66 (69.5)	64 (67.4)	81 (85.3)
10,000-19,999	73 (20.0)	58 (79.5)	51 (69.9)	48 (65.6)	65 (89.0)
20,000-34,999	88 (24.1)	65 (73.9)	59 (67.1)	56 (63.6)	75 (85.2)
≥ 35,000	109 (29.9)	88 (80.7)	77 (70.6)	77 (70.6)	93 (84.4)
**Depression score**					
High	24 (6.6)	16 (66.7)	17 (70.8)	16 (66.7)	19 (79.2)
Low	341 (93.4)	256 (75.1)	236 (69.2)	229 (67.2)	295 (86.2)
**Smoking status**					
Never	203 (55.6)	157 (77.3)	145 (71.4)	138 (68.0)	178 (87.7)
Former	97 (26.6)	72 (74.2)	68 (70.1)	65 (67.0)	83 (85.6)
Current	65 (17.8)	43 (66.2)	40 (61.5)*	42 (64.6)	53 (81.5)
**Self-rated health**					
Excellent or very good	107 (29.3)	77 (72.0)	70 (65.4)	69 (64.5)	87 (81.3)
Good	127 (34.8)	101 (79.5)	91 (71.7)	86 (67.7)	111 (87.4)
Fair or poor	131 (35.9)	94 (71.8)	92 (70.2)	90 (68.7)	116 (88.6)

* *P*<0.05 when adjusted for all other variables in table; **p<0.01 when adjusted for all other variables in table.

## Discussion

Recruitment and retention of participants, especially low income and minority members, in longitudinal weight loss trials has not proven an easy task in previous studies. Using an adaptive and comprehensive approach in BFBW, we were able to recruit a predominantly low-income minority population from local community health centers and have 86.0% complete their 24-month follow-up visit. This combination of reasonably high recruitment and high retention in this population group is unusual and we attribute our success to multiple factors [26]. First, we developed and maintained partnership relationships with health center staff, PCPs and participants which kept them connected to the study. Second, we created systems for timely tracking of recruitment and retention and monitored activities closely. Third, we used the data to generate new ideas and strategies and implemented them quickly. Specific strategies such as use of flexible part-time staff, outsourcing recruitment calls to a call center, passive PCP approval, and a willingness to meet the needs of individual participants in a variety of ways (taxi vouchers, evening and weekend appointments, allowing children at appointments, offering home visits) were most effective in achieving strong recruitment and retention numbers. Our results are consistent with Davis et al. (2002), which found that things such as providing meaningful incentives, using a participant-tracking database, maintaining between-assessment contacts, and establishing a project identity were associated with increased retention [27].

Recruitment took three months longer than our initial goal of one year. We anticipated that our study population would be approximately 66% Black and 30% Hispanic. Actual enrollment was 71.2% Black and 13.2% Hispanic. Enrollment of Blacks was as expected, but we recruited fewer Hispanics than anticipated. This is, at least in part, attributable to two administrative delays. First, Health Center C had the largest Hispanic patient population and we began enrolling participants there nearly three months after beginning at health center C, due to administrative delays. Second, we had numerous delays in translation and production of Spanish screening and study materials and did not start enrolling Spanish speakers until mid-summer 2008.

With 86.0% retention at 24-months, we exceeded our goal of 80.0%. However, retention varied by study visit. Retention was high at 6-months (74.5%), declined at 12- (69.3%) and 18-months (65.5%) and was highest at 24-months. It is unlikely that seasonal variation could explain differences in retention by visit. Participants were recruited over a 15-month period and there were participants completing each visit in every season. Additionally, 69.6% completed at least three and the majority (56.2%) attended every visit (data not shown). Differences by gender, age, race, income and randomization group may be due to a variety of factors including a predominately female staff, competing life activities for younger participants including balancing children and work, and travel. For example, it is possible that the focus of the study on weight loss and hypertension was less salient for younger participants for whom chronic disease consequences may still have been many years away [28]. We found that our Hispanic participants were more likely to leave Boston for long periods of time to visit family either in other parts of the United States or in Latin America. This may explain their lower retention at 18-months. Intervention participants were less likely to complete the 24-month visit than those in usual care. This may be due to dissatisfaction with the intervention (and its results on their weight) or fatigue (intervention participants received more contacts than usual care).

There have been few long-term weight loss interventions among low-income minority participants conducted in the United States. In a 2011 review of 38 obesity management interventions commissioned by the Agency for Healthcare Research and Quality, only four studies were conducted in predominately Black or Hispanic populations [29]. Of those studies, there were only two studies that went as long as 18-months with retention of 89.2% and 63.2% respectively. Our 24-month retention result is a major accomplishment given our setting in community health centers, and the fact that this was a pragmatic trial—we had no run in period, and had minimal behavioral inclusion criteria.

As has been noted in other studies, relationships with PCPs, administrators and staff at the health centers were key in recruiting participants [30] and maintaining high levels of retention. Nicholson et al. (2011) found that administrative support in the form of desk space, access to patient schedules and medical records, and coordination with PCPs and nurses was important for participant retention [31]. Coordination with health centers allowed us to be flexible in our strategies for recruitment and retention. For example, we could not have met with participants before or after their primary care visits if we didn’t have access to the scheduling systems, and we couldn’t have gotten some of the updates to contact information without working with the health center administrators. While there were some challenges, ultimately, having follow-up visits at the health centers was an important advantage in retaining participants and also linking the intervention to patient’s primary care.

Many attempts were needed to reach some participants, both during recruitment and retention. What worked best was spreading the calls out over time. We might call a person 10 times in the first two weeks of April, not get a response and try 10 more calls in mid-May. This strategy was effective for some participants because of out of town travel or illness. However, for others, namely the 50 people lost to follow-up, this was not an effective strategy. Our findings are consistent with work by Cotter et al. (2005) that showed that limiting contact leads to lower retention and the additional costs associated with more contact are cost-effective [32]. Kleschinsky et al. (2009) found that in a study of repeat driving under the influence (DUI) offenders that increasing calls up to 40 calls per person yielded additional completions [33]. We found that a high number of call attempts spread out over a period of months, specifically including multiple weeks in which no calls were placed, did lead to additional visit completions. We did this with approval of our IRB and with careful consideration of participant burden and ethical concerns.

It is important to put our results into context. BFBW took place during a recession. We began with an already low-income population and many faced additional hardships, including loss of jobs and homelessness during the study [34,35]. An important part of our success was the recognition that our participants led complicated lives and our study was a small, but hopefully, important part of it. We approached our participants with cultural and emotional sensitivity.

A limitation of this investigation is that we lack specific controlled information about the distinct effect of specific individual strategies implemented on recruitment and retention. Additionally we lack information on factors that have been associated with recruitment and retention in other studies such as marital status, number and age of children or other dependents, and perceived stress [8,31,36]. Future studies to more comprehensively analyze such factors might include variables such as health literacy/numeracy, social capital, satisfaction with the clinic, comorbidity, and contacts with the PCP.

## Conclusions

BFBW successfully recruited and retained predominantly minority and low-income participants at three community health centers in Boston. We learned that trials of this nature take significant and directed resources to maximize recruitment and retention. Careful tracking of recruitment strategies, with flexibility to add new strategies as required, is often needed to meet goals. Our success, along with those of several other studies shows that conducting longitudinal research in this setting is not impossible even during trying economic and political times. These are populations that disproportionately bear the burden of obesity and chronic disease and are in need of research attention. With dedication, planning, stakeholder engagement, flexibility and sensitivity, high recruitment and retention levels are achievable.

## Abbreviations

BFBW: Be Fit, Be Well; BMI: Body mass index; FDA: Food and drug administration; CHW: Community health worker; RA: Research assistant; EMR: Electronic medical record; PCP: Primary care provider; DUI: Driving under the influence

## Competing interests

The authors have no competing interests to report.

## Authors’ contributions

GGB, ETW, KME, BAR, and GAC had full access to all the data in the study and take responsibility for the integrity of the data and the accuracy of the data analysis. Study concept and design: GGB, ETW, REG, KME, and GAC. Acquisition of data: GGB, ETW, and GAC. Analysis and interpretation of data: REG, SA, KME, BAR, and GAC. Drafting of the manuscript: ETW, GGB, REG, KME and GAC. Critical revision of the manuscript for important intellectual content: GGB, ETW, REG, KME, BAR, and GAC. Statistical analysis: ETW and REG. Obtained funding: GGB, REG, KME, and GAC. Administrative, technical, and material support: GGB, ETW, SA, KME, and GAC. Study supervision: GGB and KME. All authors read and approved the final manuscript.

## Pre-publication history

The pre-publication history for this paper can be accessed here:

http://www.biomedcentral.com/1471-2458/13/192/prepub
